# Irisin enhances longevity by boosting SIRT1, AMPK, autophagy and telomerase

**DOI:** 10.1017/erm.2022.41

**Published:** 2022-12-12

**Authors:** Begoña Sánchez, Mario F. Muñoz-Pinto, Mercedes Cano

**Affiliations:** 1 Department of Physiology, Faculty of Pharmacy, University of Seville, Seville, Spain; 2 Deparment of Biochemistry and Molecular Biology, Faculty of Pharmacy, University of Seville, Seville, Spain

**Keywords:** Ageing, AMPK, autophagy, irisin, SIRT1, telomerase

## Abstract

Ageing is characterised by the accumulation of molecular and cellular damage through time, leading to a decline in physical and mental abilities. Currently, society has experienced a rapid increase in life expectancy, which has led to an increase in age-associated diseases. Therefore, it is crucial to study the process of ageing to guarantee the best conditions in the final stages of life. In recent years, interest has increased in a myokine known as irisin, which is secreted during physical exercise. This polypeptide hormone is produced by various organs, mainly muscle, and once it is released into the blood, it performs a wide variety of functions that are involved in metabolic control and may be relevant during some of the diseases associated with ageing. The aim of this review is to highlight the recent studies of irisin, such as its mechanism of expression, blood release, distribution, tissue target and participation in various cellular metabolic reactions and the relationship with key anti-ageing pathways such as adenosine monophosphate-activated protein kinase, silent information regulator T 1, autophagy and telomerase. In conclusion, irisin is a key player during the ageing process and it could be a novel target molecule for the therapeutic approach to boost longevity pathways. However, more research will be necessary to use this promising hormone for this gain.

## Introduction

Ageing is a natural biological process in which the body experiences a series of physiological and morphological alterations determined over the course of time. In a general sense, ageing implies a loss of defense capacity, reaction and adaptation, which increases the risk of suffering diseases that finally leads to death (Ref. 1).

There are various theories of ageing. One of the most accepted is the ‘free radical theory’ postulated in 1956 by the scientist Denham Harman (Ref. 2). Many free radicals are produced by the mitochondrial electron transport chain, which is necessary to produce energy. In addition to the free radicals generated in the mitochondria, for example superoxide (O^2−^), hydroxyl (OH) and hydrogen peroxide (H_2_O_2_), there are others that come from our interaction with the environment, such as solar radiation, ultraviolet light, exposure to chemotherapeutic agents and environmental toxins. However, our body has developed various genetic strategies to defend itself against the action of free radicals, the so-called endogenous antioxidant defense systems, where we find various enzymes such as superoxide dismutase (SOD), catalase, glutathione peroxidase and glutathione reductase.

During ageing there is an increase in the amount of free radicals, either because of a reduction in the protective antioxidant activity or an increase in pro-oxidant factors, all of which is reflected as an increase in cellular oxidative damage. In this way, the course of time will determine the functional decline of the organism because of histological and biochemical changes in our tissues and organs. There are numerous pathologies related to the ageing process, some of the most common in the elderly are those linked to neurodegeneration (Alzheimer's (AD), Parkinson's (PD), Huntington's diseases), metabolic disorders (obesity, diabetes), bone diseases, cardiovascular diseases, chronic inflammation or cancer.

Lifestyle plays an important role in the ageing process. A sedentary lifestyle can cause severe health problems, being one of the main risk factors related to the appearance of negative effects associated with ageing. Meanwhile, a lifestyle based on proper nutrition and physical exercise is essential for a healthy development at an old age. From a molecular point of view, this type of lifestyle stimulates anti-ageing biochemical pathways.

In this sense, during physical exercise, muscles release many myokines in response to the activity during muscle contraction. These myokines participate in several signalling pathways exerting an autocrine and endocrine effect in many tissues. Among the myokines that can be found in skeletal muscles (interleukin-6 (IL-6), IL-8, IL-15, fibrobalst grow factor 21 (FGF21) or brain-derived neurotrophic factor (BDNF)), irisin is one of the most studied because of the variety of beneficial functions it exerts. In fact, irisin could constitute a therapeutic molecule to combat metabolic disorders and age-associated diseases (Ref. 3).

The main objective of this review is to summarise the relationship between irisin and the pathways involved in longevity such as adenosine monophosphate-activated protein kinase (AMPK), silent information regulator T 1 (SIRT1), autophagy and telomerase, and their potential beneficial effect in the biological process of ageing.

## Irisin

### Biosynthesis and molecular characterisation of irisin

**Fig. 1.**
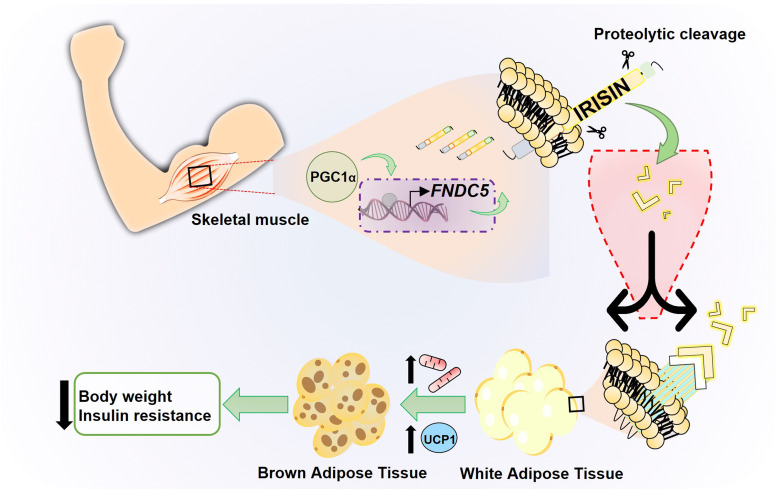
Schematic model of irisin production through physical exercise. Physical exercise leads to an increase in the transcriptional factor PGC1-*α* in skeletal muscle, which regulates the expression of the transmembrane protein FNDC5. This protein undergoes processing releasing the hormone irisin, a myokine induced by physical exercise that converts WAT into BAT, increasing thermogenesis and energy expenditure.

Irisin is a polypeptide hormone consisting of 112 amino acids with an approximate molecular weight of 12 000 Daltons (Da; 12 kDa) 100% conserved in human, mouse and rat, discovered by Boström *et al*. in 2012 (Ref. 4), who named it in honour of the Greek messenger goddess, Iris. It has been established that the release of this hormone is exercise-dependent, since the increase in physical activity stimulates, in muscle cells, the production of the peroxisome proliferator-activated receptor (PPAR-*γ*) and its transcriptional coactivator (PGC-1*α*). This results in the increase of circulating irisin (Fig. 1).

Irisin is as a product of fibronectin type III domain-containing 5 (FNDC5). FNDC5 is a type 1 transmembrane protein encoded by the Fndc5 gene and consists of 209 residues distributed in an N-terminal region (signal peptide), followed by a fibronectin III (FNIII)-like domain, a binding peptide, a transmembrane domain and the C-terminal segment. Apparently, after the insertion of FNDC5 in the membrane, cleavage occurs at the binding peptide level by an unknown proteolytic enzyme, releasing the soluble irisin into the extracellular medium. Irisin presents the typical organisation of the FNIII domain, with a *β*-sandwich folding, and the peculiarity that, unlike any other FNIII domain studied, it is structured as a dimer where the C′ chains of the four-chain sheet *β* combine to join two FNIII domains (Ref. 5). However, an irisin-specific receptor remains unknown. In some tissues, such as bone and fat, irisin acts via *α*V/*β*5 integrin receptors (Ref. 6). The flexible loops (residues 55–58 and 106–108) and the N-terminal end of the protein are located on a hydrophobic face of the dimer, suggesting that those regions are possible candidates to interact with other proteins. On the other hand, based on this dimeric structure, it has been speculated that irisin could carry out its signalling by activating and facilitating the dimerisation of an unknown receptor (Ref. 5). Therefore, these data suggest that the *α*V integrin complex is probably the main irisin receptor; however, it is important to note that this does not exclude the possibility of other irisin receptors within or outside the integrin family.

### Irisin secretion, circulating levels and metabolism

Irisin is considered as a ‘myokine’, term that refers to cytokines or other peptides that are expressed, produced and released by muscle fibres exerting their effects on other organs of the body (Ref. 7). Initially, Boström *et al*. showed that the main source of irisin was the sarcoplasm of the muscle fibre, but immunohistochemical analysis of human skeletal muscle showed that also high levels of irisin staining in the perimysium and endomysium, peripheral nerve (epineuro) and sheaths that spread between cells, sarcoplasm and subendomysium (the latter two to a lesser extent), suggesting that certain amount of irisin could come from connective tissues around skeletal muscle. This analysis also revealed the presence of irisin in several human tissues such as testicles, pancreas, spleen, brain and stomach (Ref. 8). Also, it was previously shown that irisin is expressed in salivary glands, skin, liver, kidney and heart muscle, and, interestingly, the authors conclude that cardiac muscle produces more irisin than skeletal muscle (Ref. 9). All this suggest that irisin could be produced in tissues other than muscle in an autocrine or paracrine manner.

Irisin is also secreted in white adipose tissue (WAT), so it may also behave as an ‘adipokine’. Regarding this tissue, it has been observed that in rodents irisin is secreted mainly in subcutaneous adipose tissue (SAT) adipocytes and, at a lower extent, in visceral adipose tissue adipocytes, with expression increasing when performing physical activity (Ref. 10). The fact that irisin secretion was higher in the SAT could be correlated to a greater beneficial effect of SAT compared with visceral fat.

For the circulating levels of irisin, unfortunately, there are still no confirmed reference values in rodents and in humans, since there is a high variability in the detection of circulating irisin in the different studies (Ref. 11). However, a recent meta-analysis whose objective was to determine the long-term effect of physical exercise on irisin levels in individuals of different ages and physical conditions concluded that, although there is great variability in the studies because of methodological issues and on the type of training, physical exercise had a significant positive effect on blood irisin levels in young individuals, in healthy older adults and, interestingly, in obese older adults (Ref. 12). On the other hand, a group of researchers carried out measurements of irisin levels in blood in healthy young individuals, young patients who had suffered an acute myocardial infarction and disease-free centenarians. They found that the group of disease-free centenarians showed the highest serum irisin levels (35.3 ± 5.5 ng/ml) compared with young healthy individuals (20.7 ± 6.3 ng/ml) and especially in comparison with young patients with acute myocardial infarction (15.1 ± 5.4 ng/ml). Interestingly, the group of centenarians studied did not have serious diseases related to ageing (such as diabetes, cognitive failure, cancer, heart attacks, kidney or liver failure or physical disabilities) (Ref. 13). These data seem to indicate that circulating irisin levels could be significantly associated with prosperous ageing free of associated diseases. These same researchers carried out a study of two single-nucleotide polymorphisms (SNPs) of the FNDC5 gene to investigate whether there were differences in irisin activity between both SNPs in healthy centenarians and healthy controls (20–81 years, from various populations; Spain, Italy and Japan). However, no differences were found between the genotypic/allelic frequencies of the two SNPs in centenarians versus controls in either population, nor a significant association between the two SNPs and successful disease-free longevity (Ref. 14).

Another study revealed that basal irisin was higher in young adults than in older adults and that circulating irisin increased immediately after high-intensity interval exercise, but not in chronic exercise, and this exercise-induced irisin secretion was independent of age or fitness level (Ref. 15). This suggests that perhaps other factors, such as levels of physical exercise throughout life, nutritional habits or complex genetic and environmental associations that have not yet been determined, could have a stronger influence on irisinaemia. Also, it could suggest that there is a mechanism of autoregulation of irisin levels when chronic exercise is practiced avoiding a possible dysregulation of metabolic pathways. In any case, more research would be necessary in this regard.

In relation to the pharmacokinetics of irisin, a short half-life (1 h) of injected recombinant irisin in mice has been reported (Ref. 6), but there is little information on its metabolism and elimination. The administration of irisin in vivo in rodents revealed that the hepatobiliary and renal systems act together to guarantee the correct excretion of the hormone. In this experiment, 125I-irisin was tracked in mice after intravenous injection using single-photon emission computed tomography and, 15 min after the injection, irisin levels were observed in the gallbladder, liver and kidneys, decreasing its concentration between 30 and 120 min. The joint action of the hepatobiliary–renal system seems to be because of the molecular weight of irisin; when peptides heavier than 10 kDa are not completely metabolised at the renal glomerular filtration level, then the hepatobiliary system comes into play as reinforcement (Ref. 16). Furthermore, an earlier study showed a decrease in serum irisin levels that coincided with an increase in enzymes in the liver (Ref. 17). Even so, further investigation is required on whether the mechanism of irisin elimination through the hepatobiliary system involves the participation of enzymes.

### Target tissues and mode of action of irisin

Irisin, as a hormone, allows a crosstalk between the muscle and other organs, including adipose tissue, bone, brain, liver, pancreas, vascular bed, heart and brain, as well as communication within the muscle itself (Ref. 18). Table 1 summarises the pleiotropic effect of irisin on its different target tissues and the pathways involved, highlighting the paths of longevity that will be discussed in the Section ‘Irisin and longevity pathways’.

**Table 1.** Irisin target tissue, signalling pathways and its beneficial effect

Target cells/tissue	Signalling pathway	Effects	References
Muscle	AMPK/pAMPK SIRT1 PI3K/Akt	Enhance glucose uptake Improve insulin sensitivity	24, 25, 26, 56, 57 70, 71, 73
Adipose tissue	P38MAPK	Browning of WAT Improving metabolism profile Inhibit adipogenesis	4, 21, 22, 23
Pancreas	AMPK	Decrease NEFA and triglycerides Inhibit the growth of pancreatic cancer cells	60
Cardiovascular system	ERK/MAPK NO AMPK Autophagy	Endothelial cell proliferation Decrease arterial pression Protection of cardiomyocytes Protecting cardiac hypertrophy Protect from damage following myocardial infarction Improve insulin sensitivity in myocytes	25, 26 63, 64, 65 89, 91 90 91 92
Liver	Telomerase	Improved mitochondrial function in hepatocytes	93
Bone	ERK/MAPK	Osteoblast proliferation and differentiation	25, 26, 27, 28
Kidney	MAPK	Decreased reactive oxygen species (ROS) and apoptosis	62

One of the most important target tissues of irisin is adipose tissue. There are key differences within its two types. Although WAT is the main energy storage resource in the form of lipids (mainly triglycerides) agglomerated in a large droplet in adipocytes, brown adipose tissue (BAT) metabolises fatty acids, releasing energy in the form of heat. BAT can perform its function thanks to mitochondrial uncoupling protein 1 (UCP-1), also called thermogenin. It has been described that exercise-mediated increase in UCP-1 leads to WAT ‘browning’, a process in which adipocytes adopt a more BAT-like phenotype. Browning is associated with an increase in energy expenditure and therefore favourable effects on metabolism (Refs 19, 20). Boström *et al*. (Ref. 4) showed that irisin was involved in mediating the PGC-1*α*-induced browning of WAT through upregulation of UCP-1 expression in adipocytes, partly via increased PPAR-*α* expression (Fig. 1). Therefore, irisin functions as a muscle-derived molecule that communicates directly with adipose tissue acting as a thermogenic protein by increasing UCP-1 levels and promoting WAT browning. This effect improves the metabolic profile of the body, making irisin a potential new target for the treatment of metabolic diseases.

In this regard, genistein-treated mice directly increased circulating levels of irisin, which stimulated the browning process of adipose tissue, increasing energy expenditure (Ref. 21). Also, cold exposure increases the UCP-1 levels the same way as physical exercise, by increasing irisin levels. In an experiment where recombinant irisin was introduced into primary adipocytes and mature adipocytes, irisin was found to mediate the increase of UCP-1 through signalling pathways involving p38 mitogen-activated protein kinase (p38MAPK) and p38MAPK kinases related to extracellular signals (ERK). Irisin induced phosphorylation of p38MAPK and ERK, which leads to an increase of UCP-1, effect that can be suppressed by p38MAPK (SB203508) and ERK (U0216) inhibitors (Ref. 22). Furthermore, experiments in adipocytes cultures have shown that irisin can suppress adipogenesis by inducing Wnt signalling, which subsequently inhibits transcription factors responsible for the formation of mature adipocytes (Ref. 23).

Another important role of irisin is the maintenance of muscle facilitating glucose uptake and improving its insulin sensitivity. Thus, irisin augments insulin-induced phosphatidylinositol 3-kinase (PI3K)/Akt signalling activity which leads to the increase of glucose inflow to the muscle cell by promoting glucose transporter type 4 translocation to the membrane (Ref. 24). Other studies have shown that FNDC5 overexpression improved insulin resistance via AMPK pathway (Ref. 25). At the systemic level, irisin maintains glucose and lipids homoeostasis which is crucial in treatment for type 2 diabetes mellitus (T2DM), obesity or other metabolic diseases (Ref. 26).

**Fig. 2.**
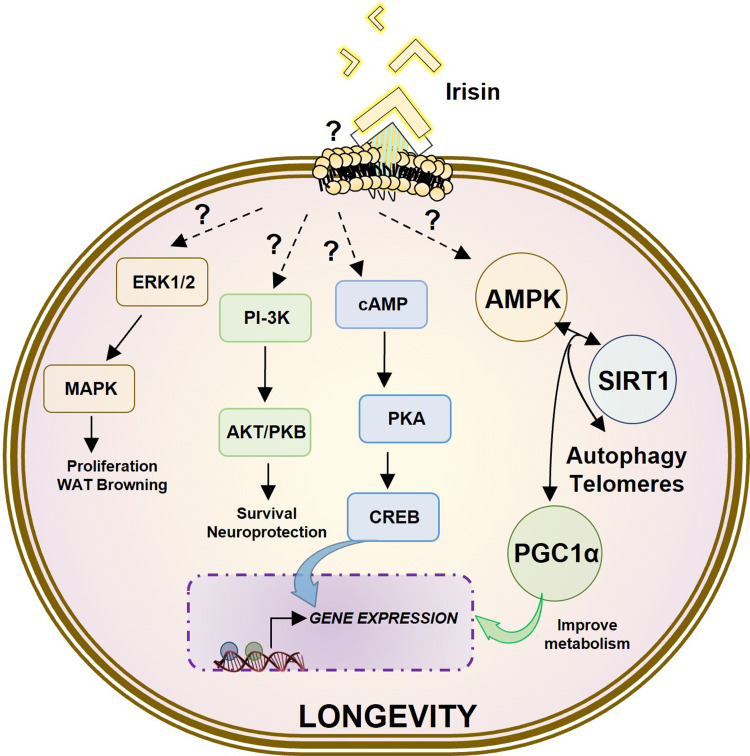
Schematic representation of the main signalling intracellular pathways activated by irisin. Irisin induces several cellular responses such as WAT browning, proliferation, neuroprotection, survival and longevity through the activation of ERK1/MAP-kinase, PI3K/AKT/Protein kinase B (PKB), AMP/PKA/CREB, AMPK/SIRT1/PGC*α*, autophagy and telomerase pathways.

Recent studies have shown that FNDC5/irisin acts through MAPK signalling pathways in numerous cellular processes, such as neural differentiation, endothelial cell proliferation as well as osteoblast proliferation and differentiation (Refs 25, 27) preventing bone loss by p38 and ERK activation (Ref. 28) (Fig. 2).

Therefore, irisin exerts its effects through different pathways that summarise several of the favourable effects of physical exercise, including white adipocyte browning, neural proliferation, bone metabolism or glucose homoeostasis. This may represent a defense against pathological states often related to ageing, such as obesity, diabetes, hepatic steatosis, osteoporosis or neurodegenerative diseases. The latter, because of its importance as a disease associated with ageing, will be discussed in the following section.

### Irisin in neurodegenerative disorders

It has been reported that exercise is involved in many physiological processes at the central nervous system that may improve memory, attention and promoting neuronal plasticity even in elderly patients, being a non-pharmacological approach to prevent the brain ageing vulnerable to AD, PD and stroke (Ref. 29).

Recently, studies have shown that myokines released by skeletal muscle are responsible for those beneficial effects, although its mechanisms have not been fully identified yet (Ref. 30). Some authors suggest that irisin released in the blood stream by the muscle can reach the brain inducing BDNF expression (Ref. 31). However there are some gaps in the irisin mechanism of action that must be addressed in further studies. For example, it is not well understood how irisin can cross the blood–brain barrier, despite that irisin circulating levels in cerebrospinal fluid (CSF) are age-related increased in healthy humans even in elderly (Refs 32, 33). Studies in mice showed that irisin can induce anti-apoptotic signals in response to cerebral ischaemia mediated by AKT and ERK1/2 activation (Ref. 34) and activate cAMP–protein kinase A (PKA)–cAMP response element-binding (CREB) pathway, which has been shown to participate in neural differentiation and neuroprotection (Ref. 35). The activation of this pathway mediated by irisin enhances BDNF, rescuing the synaptic plasticity in a model of AD (Ref. 36). Taken together, these results suggest that irisin could have an important role as neuroprotector (Fig. 2).

The potential of irisin in AD has been described both in vivo and in vitro. In vitro studies showed that irisin-supplemented media from astrocytes decreased *β*-amyloid toxicity also decreased the levels of proinflammatory cytokines (IL-6, IL-1*β* and cyclooxygenase-2 (COX-2)) (Ref. 37). Some authors suggest that irisin can bind a specific domain between *β*- and *α*-secretase cleavage sites of amyloid precursor protein (APP) reducing the amyloid beta-peptide (A*β*) formation of both A*β*40 and A*β*42 peptides (Ref. 38). In macrophages stimulated with lipopolysaccharide ameliorated the inflammatory signalling decreasing the levels of TLR4, MyD88 and NF-*κβ* (Ref. 39). In vivo studies, in a mouse model of AD, exercise-induced adult hippocampal neurogenesis associated with an increase of BDNF and irisin (Ref. 40); however, it is not fully understood how irisin enhances BDNF expression in the hippocampus (Ref. 41). In mice with genetic deletion of irisin cognitive function is impaired during exercise, ageing and AD (Ref. 42). In addition, irisin levels were found reduced in CSF of AD mice and patients. These authors showed how decreasing the levels of irisin in mice brain impairs cognitive behaviour that were restored by increasing the peripheral expression of irisin (Ref. 36). Regression analyses of plasma levels and CSF of irisin revealed that there is a positive relationship between irisin levels and cognition (Refs 43, 44). However, the correlation loss was significant in patients with (Ref. 44) AD.

Few studies reported that irisin could have a beneficial effect on other neurodegenerative disorders. In an MPTP (1-methyl-4-phenyl-1,2,3,6-tetrahydropyridine), animal model of PD, authors showed that rats treated with irisin and bone marrow stromal cells (BMSCs) prevented the dopaminergic neurons loss and irisin facilitated the stem cells with regenerative ability inducing the migration of BMSCs in the damaged brain area (Ref. 45). In a different animal model of PD (6-hydroxydopamine (6-OHDA)), authors demonstrated that irisin induced by exercise (treadmill running for 16 weeks), before disease induction, prevented motor and cognitive behaviour dysfunction (Ref. 46). Irisin also prevented the loss of dopamine neurons and striatal dopamine levels in an α-synuclein preformed fibrils (*α*-syn PFF)-induced model of PD. The authors suggested that irisin promotes the endolysosomal degradation of pathological *α*-syn (Ref. 47). Regarding the amyotrophic lateral sclerosis (ALS) the results seem to be contradictory. Some authors showed that serum irisin levels were upregulated in ALS patients (Ref. 48), meanwhile in animal models with mutant SOD1, levels of irisin and myostatin have found downregulated (Ref. 49). This discrepancy could be related to the interspecies variability or the fact that the muscle damage produced in ALS patients increases irisin to carry on its anabolic action on skeletal muscle (Ref. 48). Further studies must confirm this evidence, but it could be possible the use of irisin as a biomarker of muscle damage in neurodegenerative diseases.

All these facts support the idea that irisin could be the linkage molecule between brain and skeletal muscle that exert its beneficial effects through BDNF expression. Evidence suggests that the sole injection of irisin in mice could even substitute the exercise itself without the loss of its potential benefit (Ref. 50). This would provide a major stride in elderly patients with restrained mobility capabilities.

## Irisin and longevity pathways

Several modulators of longevity and health span have been extensively validated. Among others these include four well-studied pathways that are known to regulate ageing, and whose modulation has been shown to influence the rate of ageing, these are AMPK, and sirtuin pathways, autophagy and telomere length (TL) (Refs 51, 52, 53, 54). In this section, we will discuss the relationship between each of these pathways of longevity and irisin, trying to summarise the relations of irisin and these pathways and their beneficial effects on longevity. Figure 2 illustrates how irisin converge, among others mentioned above, in an enhancement of the paths of longevity.

### Irisin and AMPK

AMPK is a highly conserved enzyme in the eukaryotic kingdom that is activated when energy levels are low, influencing a wide range of physiological processes with the purpose to increase energy production and coordinate a decrease in the expenditure of adenosine triphosphate (ATP). This protein is formed as a heterotrimeric complex composed of a catalytic subunit (*α*) and two regulatory subunits (*β* and Y). Its activity is controlled by kinase-type enzymes that carry out phosphorylation activation by a competitive nucleotide binding mechanism. At low-energy levels, the AMP/adenosine diphosphate (ADP):ATP ratio increases, favouring the binding of AMP/ADP, which determines a high activation of AMPK. The attractiveness of AMPK as a promoter of healthy ageing is based on its ability to integrate multiple signalling and transcription pathways that are known to favour longevity (mitochondrial biogenesis, autophagy, beta-oxidation or glucose uptake, among others) (Ref. 55).

The energy demand by the muscle produced during physical exercise potently activates AMPK, and it has been seen that this protein is regulated by various skeletal muscle myokines; among them, irisin has been shown to induce AMPK phosphorylation and activation in vitro (Ref. 15). The researchers measured intracellular ATP in human skeletal muscle cells treated with irisin and found a significant decrease in the levels of this molecule after 1 h, this decline coincided with the increase in AMPK phosphorylation. To confirm that the effect was because of irisin, cells were treated with an AMPK inhibitor and it was found that irisin-induced AMPK phosphorylation and regulation of metabolic genes were effectively prevented under these conditions. In this way, the decrease in the amount of ATP that determines a low-energy level may be the trigger for irisin-mediated AMPK phosphorylation and its consequent effects on cellular metabolism. Again, a study linking the role of irisin in hypoglycaemic situations with AMPK function in differentiated muscle cells and confirmed that irisin treatment increased AMPK phosphorylation (Ref. 56).

Another research group concluded that skeletal muscle AMPK is necessary to maintain basal FNDC5 expression, by comparing AMPK gene knockout mice with wild-type mice and observing a drastic decrease in FNDC5 expression in the first group of mice. Likewise, in wild-type mice, AMPK phosphorylation is increased immediately when muscle contraction occurs, an effect that was abolished in knockout mice (Ref. 57). Moreover, a study demonstrated that AMPK pathway is involved in the PGC-1*α*/FNDC5 and irisin expression using a flavonoid, icariin, which enhances dose-dependent phosphorylation of AMPK protein and increased mRNA levels of irisin/FNDC5 in C2C12 myocytes and decreased body weight gain in mice (Ref. 58). Similar effects have been found in rats treated with the flavonoid ampelopsin which attenuates the atrophy of skeletal muscle through activating AMPK/SIRT1/PGC-1*α* signalling cascade (Ref. 59).

**Fig. 3.**
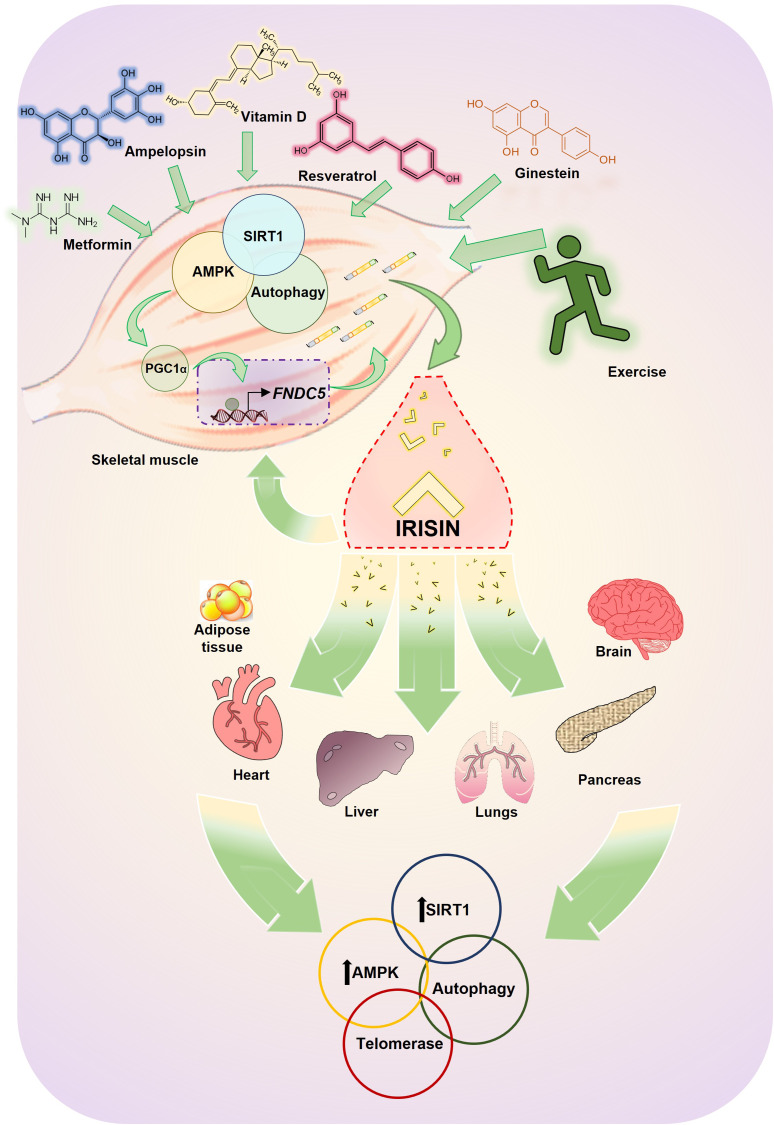
Schematic representation of irisin's effect on molecular longevity pathways. Irisin levels increase after exposing to exercise or several pharmacological or nutraceutical treatments. The irisin released by the muscle has an autocrine effect on the muscle itself and an endocrine effect on many tissues such as adipose tissue, heart, liver, pancreas, lung or brain, improving metabolism and function. Between the pleiotropic effects of irisin some of them are mediated by stimulation of longevity pathways such as AMPK, SIRT1, autophagy and telomerase.

All these experiments show how the secretion of irisin by the muscle depends on AMPK signalling and, in turn, irisin has an autocrine effect, increasing the AMPK pathway and thus improving the metabolism and function of the muscle itself (Fig. 3).

On the other hand, different studies confirm how irisin can improve the functionality of various tissues in an endocrine way by stimulating the AMPK pathway. Thus, in rat insulinoma cell line (INS-1E) models for insulin secretion regulation and pancreatic islet beta-cell function studies, irisin reversed the intracellular accumulation of non-esterified fatty acids (NEFAs) and triglycerides induced by glucolipotoxicity through the activation of AMPK signalling. Irisin also suppressed overnutrition-induced inflammation in INS-1E cells, providing scientific basis that irisin has potential benefit in pancreatic *β*-cells (Ref. 60). Moreover, irisin has also been seen to inhibit the growth of pancreatic cancer cells in a dose-dependent manner by inhibiting the mammalian target of rapamycin (mTOR) pathway and activating the AMPK signalling (Ref. 61).

In this sense, the AMPK pathway is involved in the action mechanism of irisin in other tissues. Thus, some findings revealed that aerobic exercise participates in alleviating the levels of oxidative stress and apoptosis in impaired kidney tissue following myocardial infraction in part because of activation of the FNDC5/irisin–AMPK–SIRT1–PGC-1*α* signalling pathway (Ref. 62). At the vascular level, in spontaneously hypertensive rats, it was shown that irisin injection reduced blood pressure through the AMPKAkt–endothelial NO synthase (eNOS)–nitric oxide (NO) signalling pathway, increasing the release of NO (Ref. 63). A similar effect was obtained in another similar study with obese mice that showed endothelial function improvement after injection with irisin (Ref. 64). Also, in hypoxia/reoxygenated injury-induced endoplasmic reticulum stress irisin has a protective effect on cardiomyocytes in an AMPK-dependent manner (Ref. 65).

Interestingly, although irisin levels were not measured, experiments with rats performing swimming exercises showed restoration in the survival pathways pAMPK/SIRT1/PG1-*α* in the hippocampus in an induced ageing-dependent manner after 8 weeks of training demonstrating again the relations between exercise and the enhancement of the survival pathways (Ref. 66).

In short, the AMPK pathway is one of the promoters of healthy ageing through the regulation of various aspects of metabolism. In this context, the activity and regulation of AMPK are integrated into a complex feedback network in which molecules such as PGC1-*α*, SIRT1 and irisin participate. With the performance of physical exercise and the consequent increase in irisin levels, the activation of AMPK and its subsequent positive effects on energy metabolism could be promoted, which results in a favourable process.

### Irisin and SIRT1

SIRT or sirtuins are a family of NAD+ dependent proteins, which were initially identified as class III histone deacetylases (Ref. 67). Later, it was discovered that they were essential regulators of the control network of energy homoeostasis and participated in a wide number of cellular processes (such as glucose and fat metabolism), exerting their action on numerous nuclear transcriptional factors and on specific cytoplasmic and mitochondrial proteins. In mammals, this family encompasses from SIRT1 to SIRT7, all of them presenting a conserved catalytic domain but with certain differences according to tissue specificity, subcellular location, enzymatic activity and targets (Ref. 68). Within this family, the most studied enzyme is SIRT1, a protein that is expressed mainly in the nucleus, although under certain specific conditions it can act on cytosolic proteins (Ref. 69).

SIRT1 is of great interest in this study since one of its non-histone targets is PGC1-*α*, which undergoes reversible acetylation/deacetylation control; when deacetylated by SIRT1, PGC1-*α* is activated, which leads to the regulation of subsequent pathways involved in the control of mitochondrial gene expression and the increase in activity of PGC1-*α* that, in muscle, results in an increase in circulating irisin. PGC1-*α* is a major regulator of glucose homoeostasis, lipid catabolism, biogenesis and mitochondrial function. The signalling pathway of this transcriptional co-activator is associated with the mitochondrial antioxidant system, and in turn, SIRT1 prevents the loss of mitochondria and modulates DNA damage responses. SIRT1 promotes protective pathways such as the one carried out by PGC1-*α*, so both may be good candidates to prevent and/or treat diseases related to health or ageing processes (Ref. 68).

Extrapolating this situation to the ageing process, where there is a decline caused mainly by the presence of free radicals and oxidation, may show that SIRT1, an important anti-ageing factor, performs its protective role by acting together with PGC1-*α* and activating pathways of antioxidant protection, where irisin may be involved. Thus, in this revision we are interested in the link between irisin and SIRT1 (Fig. 2).

Some studies have investigated the effect of exercise on SIRT1 expression and activity. Thus, in aged rats, SIRT1 activity increased in heart and adipose tissue after a 6 weeks treadmill training programme (Ref. 70). Also, muscle biopsy of male cyclist after 3 days of exhaustive training showed both acute and chronic elevations in PGC-1*α* and SIRT1 mRNA expression (Ref. 71). In addition, in rats undergoing swimming training the signalling pathway of AMPK/SIRT1/PGC-1*α* was enhanced in the hippocampus (Ref. 66).

In this regard, in experiments with an animal model for acute lung injury irisin pretreatment not only significantly augmented the ratio of p-AMPK/AMPK protein, but also up-regulated the expression of SIRT1 protein in lung tissue improving the alveolar epithelial barrier dysfunction (Ref. 72). Moreover, irisin and sirtuin-1 mRNA were investigated in the soleus muscle tissue of diabetic rats that were trained by running on a treadmill for 12 weeks. The researchers found a significant increase in the expression of these genes, which can be a compensatory mechanism for reducing oxidative stress and improving the symptoms in diabetics (Ref. 73).

Interestingly, there are some clinical and preclinical studies about drug-induced stimulation or nutraceutical/phytotherapic interventions in irisin secretion and some of them show the involvement of the SIRT1 pathway (Ref. 74). Thus, metformin, a widely used drug of T2DM that improves insulin sensitivity and inhibits hepatic gluconeogenesis (Ref. 75), can promote up-regulating irisin expression in mice muscle (Ref. 76) and in INS-1E cells in high-glucose environment through AMPK/SIRT1/PGC-1*α* signal pathway, improving its function and survival (Ref. 77).

Resveratrol (3,5,4′-trihydroxystilbene), a phenolic compound found in grapes that modulates various processes related to ageing (such as oxidative damage, inflammation, telomere wear and cellular senescence) and a powerful activator of the sirtuin family, especially SIRT1 (Ref. 78), was also studied in relation to irisin. Resveratrol administered orally in mice and in humans determined an increase in the expression of FNDC5 and UCP-1 in SAT and improves the glycaemic and lipid profiles. In this study it was shown that the candidate molecule that would mediate the relationship between the administration of resveratrol and the increase in FNDC5 and UCP-1 would be SIRT1 (Ref. 79). However, other experiments found that rats treated with resveratrol or grape juice increased the expression of genes FNDC5/UCP-2 in muscle but not in adipose tissue (Ref. 80) (Table 2).

**Table 2.** Irisin inducer that have been studied in relation to longevity pathways

Inducers	Pathway boosted	Target cells/tissue	Effects	References
Icariin	AMPK	C2C12 myocytes, mice	Decrease body weight gain	57
Ampelopsin	AMPK/SIRT1/PGC-*α*	Rat skeletal muscle	Attenuate atrophy	58
Genistein	AMPK	Adipose tissue	Browning	21
Metformin	SIRT1	INS-1E cells Obese mice	Improve pancreatic cells survival	74, 75
Resveratrol	UCP-1, SIRT1	Muscle, adipose tissue	Improve the glycaemic and lipid profiles	78, 79
Vitamin D	SIRT1	human	Decrease glycosylated haemoglobin	80
Omega-3		Diabetic human	No results yet	81
Cardamom		Diabetic human	No results yet	82

In the same sense, a clinical trial with 90 men and women with type 2 diabetes and overweight/obesity who were supplemented with vitamin D showed that there was a parallel significant increase in the plasma levels of irisin and SIRT1 supporting the link between these two molecules, considering that SIRT1 stimulates PGC-*α* which in turn enhances irisin production (Table 2).

Currently, there are other similar clinical trials. One is studying the effect of omega-3 fatty acids supplementation on irisin and SIRT1 levels in diabetic patients treated with glimepiride (Ref. 81). And, the other one studies the effect of cardamom supplementation not only on lipid profile, oxidative stress or blood glucose but also in SIRT1 and irisin plasma levels in T2DM patients (Ref. 82).

In conclusion, the metabolic sensor SIRT1 is one of the gatekeepers that control the activity of PGC1-*α*, and together they determine an essential link in the regulatory network of metabolic homoeostasis. Therefore, the increase in PGC1-*α* determines the increased expression of FNDC5 and consequently the release of irisin. However, irisin can also activate the SIRT1 pathway in many tissues. In fact, it seems that SIRT1/PGC1-*α* and FNDC5/irisin can potentiate each other and the relationship between them could explain many of the protective effects related to physical activity.

### Irisin and autophagy

Autophagy is a highly conserved catabolic process in eukaryotes that is activated under conditions of nutrient scarcity, and results in the renewal of cytoplasmic material as well as the mobilisation of macromolecules to generate highly energetic compounds that cover cellular demands under conditions of decreased internal or external resources (Ref. 83). Autophagy is essential for the maintenance of cellular homoeostasis, improving the turnover of organelles and non-functional proteins, being essential in neuronal cells as a method of neuroprotection in response to stress. All neurodegenerative diseases share certain pathogenic mechanisms, including age-associated autophagic flux imbalance that causes an inability to remove neurotoxic oligomers or misfolded proteins (Ref. 84). Numerous studies show that autophagy promotes longevity in model organisms, and although this role is not fully established in mammals, it is known that an increase in autophagy is an effective mechanism to prevent premature ageing, improve health and promote longevity in mammals (Ref. 83).

The regulation of autophagy involves pro-ageing molecules such as mTOR and insulin-like growth factor-1 (IGF-1), as well as anti-ageing molecules, such as SIRT and AMPK. AMPK has been recognised as a key activator of autophagy through a mechanism that involves mTOR inhibition (under starvation conditions, or when the AMP/ATP ratio is raised) and activation by phosphorylation of FOXO-3 and the Ser/Thr protein-ULK-1 kinase, proteins involved in the development of the autophagic process (Ref. 85). In contrast, mTOR is the primary inhibitor of autophagy in both animal models and humans; IGF-1 signals through its IGF1-R receptor that activates mTOR to inhibit autophagy. For its part, SIRT1 and its orthologues also participate in the regulation of autophagy as activators of the process, by the deacetylation of certain genes belonging to the atg family (an important group of proteins involved in autophagy) and other transcription factors (p53, NFK-*β*, FOXO-1, -3, -4 and PGC1-*α*) (Ref. 86).

Some of the beneficial effect of physical activity seen to be related to autophagy induction in many tissues including muscle, adipose tissue, liver, pancreas, heart or brain (Refs 87, 88). Considering the pleiotropic effects of irisin recent studies have focused on the relation between the FNDC5/irisin system and the autophagy process mediating the beneficial effects of exercise.

Recently, studies with hypertrophic heart models comparing wild-type mice with FNDC5 knockout mice showed that irisin deficiency caused a decrease in autophagy and aggravated autophagic failure, whereas exogenous supply of irisin induced autophagy and improved the development of the process. The activation of autophagy was mediated by irisin by activating AMPK, which carried out an activating phosphorylation on ULK-1. In this experiment, markers LC3-II (number of autophagosomes) and p62 (protein that is degraded in the process and serves to determine autophagic flow) were measured; the supply of exogenous irisin increased the levels of LC3-II and decreased those of p62, that is, irisin promotes autophagy and autophagic flow protecting against pressure overload-induced cardiac hypertrophy (Ref. 89).

In studies on hypoxia-treated cardiomyocytes in vitro and on infarcted hearts in vivo irisin was capable to enhance autophagy favouring the elimination of the damaged mitochondria and protect them from further damage following myocardial infarction, resulting in an increase in ATP levels in response to hypoxia (Ref. 90).

In another experiment, an attempt was made to evaluate whether irisin regulated autophagy and autophagy-mediated apoptosis in cardiomyocytes with hypertrophic damage induced by angiotensin-II (Ang-II). Ang-II increased the levels of LC3-II and p62, which indicates an increase in the level of autophagy but a flow blockage, that is, although there is an increase in autophagy, the process is failing by not being resolved appropriately. Exogenous irisin supplementation reduces p62 levels and increases LC3-II levels, promoting increased autophagy and autophagic flux. In addition, an increase in the number of autophagosomes but not of autophagolysosomes was observed in cardiomyocytes with Ang-II. Also, when applying irisin, an increase in both autophagosomes and autophagolysosomes was observed, which translates into correct functioning and resolution of the autophagic mechanism (Ref. 91).

Other studies in C2C12 myoblast cells showed that irisin induced reversion of insulin resistance through induction of autophagy. Thus, C2C12 cells augmented the autophagic rate increasing the level of the autophagosome marker LC3, and increased p62 degradation (Ref. 92).

Interestingly, hepatic ischaemia–reperfusion (IR) of old rats treated with exogenous recombinant irisin showed higher level of p62 and LC3BII proteins and higher amount of autophagosomes improving inflammation, oxidative stress, apoptosis and liver injury in an old rat model of hepatic IR (Ref. 93).

Although it is necessary to continue investigating the role of irisin in autophagy, it seems that this myokine has a potential to link the molecular mechanism of exercise with its beneficial effect on diseases and ageing (Fig. 2).

### Irisin and telomeres

Telomeres are the protective DNA structures found at both ends of eukaryotic chromosomes, whose role is to protect, maintain the integrity and preserve the information of our genome.

Telomeres progressively shorten with each cell division, which leads to senescence, apoptosis or oncogenic transformation of somatic cells, affecting the health and life expectancy of the individual. That is why telomere length (TL) is considered as a ‘molecular clock’ that determines the state of ageing of the organism. The rate of telomere shortening can increase or decrease under the influence of lifestyle, so that a correct diet and physical exercise can reduce the rate of shortening or at least prevent excessive telomere wear (Ref. 94).

Using TL as an ageing marker, Rana *et al*. investigated in 2014 the association between body composition, plasma irisin levels and TL in peripheral blood mononuclear cells from healthy, non-obese individuals. In this study the participants did not perform any type of training 12 h before the analysis, and the results showed no correlation of TL with anthropometric or inflammatory parameters; however, TL has a significant negative correlation with age and a significant positive correlation with plasma irisin levels; the greater the amount of irisin present in the individual, the greater the TL. These results determine that irisin is a hormone with anti-ageing properties, although the precise mechanism by which irisin promotes telomere lengthening is unknown. The authors suggest that this effect can be mediated by irisin activating the p38MAPK pathway, which regulates the expression of telomerase reverse transcriptase (Ref. 3). However, the ability of irisin to enhance telomerase expression and its precise mechanism warrants further investigation.

Finally, in a model of hepatic IR irisin improved mitochondrial function via increasing telomerase activity in rat aged hepatocytes. Inhibition of telomerase activity by inhibitor BIBP1532 abolished the protective role of irisin in hepatocytes during hypoxia and reoxygenation (Ref. 93).

Although more studies are needed regarding the possible effects of irisin on telomerase activity, it appears that irisin exerts part of its potentially anti-ageing effects through this mechanism (Fig. 2).

## Conclusions and future perspectives

Physical exercise can be recognised as a non-pharmacological strategy to counteract dysfunctions associated with age. Part of these effects could be mediated by irisin signalling, through the activation of other molecules and cellular processes associated with the promotion of healthy ageing and increased life expectancy, such as AMPK, SIRT1, telomere elongation and autophagy, both in an autocrine and endocrine manner. Scientific evidence shows that irisin performs its role in various tissues such as the cardiovascular system, adipose tissue, liver, skeletal muscle, pancreas or brain, participating in metabolic processes responsible for maintaining cellular homoeostasis and stimulating anti-ageing signalling pathways, which would promote beneficial longevity with fewer diseases (Fig. 3).

Most of these studies have been done in preclinical models. However, clinical trials carried out so far have been designed to evaluate the effect of physical exercise on irisin levels and its benefit in unhealthy situations, many of them in age-related diseases such as type 2 diabetes, overweight/obesity, metabolic syndrome or cardiometabolic disease (Refs 95, 96, 97). Other trials focus on the study of supplementation with substances known for their anti-ageing effect, such as metformin (Ref. 98), resveratrol (Ref. 79) or vitamin D (Ref. 99), on irisin secretion and the latter also on levels of SIRT1. But there are few clinical trials studying irisin on longevity. In this regard, one of them compared circulating irisin after an acute episode of circuit training in overweight young adults and older adults and both showed a similar irisin response (Ref. 100). Likewise, a clinical trial with healthy centenarians, healthy youth and myocardial infarction patients found higher serum irisin levels in disease-free centenarians (Ref. 13), suggesting that high irisin levels may be related to healthy longevity in humans. However, there are no clinical trials to assess the effect of irisin on the longevity pathways that are the subject of this review, such as AMPK, SIRT1, autophagy or TL. For that, more research is expected in the future to test the potency of irisin on human longevity. Thus, it would be necessary to carry out clinical trials to measure the effect of irisin on markers of longevity such as SIRT1, AMPK, autophagy and TL, as well as other markers of ageing such as inflammatory mediators (IL-6, protein C reactive, tumour necrosis factor-*α*, etc.), sarcopaenia index, frailty index or cognitive tests in older adults and centenarians, both healthy and sick, and carry out interventions with different types of training (acute, chronic, aerobic, bodybuilding or exercise intensity level), trying to understand what type and frequency of exercise is the one that reports the highest amounts of irisin.

Although some preclinical studies have been conducted in rodents to evaluate the administration of irisin as a pharmacological strategy (Ref. 74), it would be very interesting to carry out clinical trials to assess the effect of the administration of irisin in human of different ages but especially in advanced ages to evaluate its possible pharmacological effect against several diseases and disorders.

On the other hand, the main limitation that currently exists in irisin studies is that there are low correlations between the levels of irisin measured with different enzyme-linked immunosorbent assays (ELISAs) and even rather low correlations between the values determined by ELISA and mass spectrometry (Refs 11, 101, 102), so it seems that no reliable and reproducible method is yet available for the measurement of irisin. In fact, different meta-analyses have reported decreased irisin values in patients with diseases such as coronary heart disease (Ref. 103) or osteoporosis (Ref. 104), but the reported irisin levels were highly variable, from pg to μg/ml, which limits their validity. In addition, to the problems of detection of irisin plasma levels, more research on the pharmacokinetics and pharmacodynamics of irisin would also be necessary.

Another interesting aspect of irisin is the potential use in preventive/therapeutic strategies, thanks to the possibility of using natural compounds and various drugs that can modulate the levels of this myokine and that could interact with the longevity signalling pathway (Table 2). However, many aspects remain to be clarified and investigated in this regard.

In conclusion, as this revision suggests, irisin is a key player during the ageing process and it could be a novel target molecule for the therapeutic approach to boost longevity pathways. In the next few decades, researchers will face the challenge of seeking anti-ageing strategies that will enable us to find healthy and disease-free ageing and irisin could be a likely agent, but more research will be necessary to overcome some controversies raised regarding the use of this promising hormone in ageing.
